# High phylogenetic diversity is preserved in species-poor high-elevation temperate moth assemblages

**DOI:** 10.1038/srep23045

**Published:** 2016-03-16

**Authors:** Yi Zou, Weiguo Sang, Axel Hausmann, Jan Christoph Axmacher

**Affiliations:** 1 UCL Department of Geography, University College London, London, UK; 2 Centre for Crop Systems Analysis, Wageningen University, Wageningen, The Netherlands; 3 College of Life and Environmental Science, Minzu University of China, Beijing, China; 4 The State Key Laboratory of Vegetation and Environmental Change, Institute of Botany, Chinese Academy of Sciences, Beijing, China; 5 Lepidoptera Section, Bavarian State Collection of Zoology, Munich, Germany

## Abstract

Understanding the diversity and composition of species assemblages and identifying underlying biotic and abiotic determinants represent great ecological challenges. Addressing some of these issues, we investigated the α-diversity and phylogenetic composition of species-rich geometrid moth (Lepidoptera: Geometridae) assemblages in the mature temperate forest on Changbai Mountain. A total of 9285 geometrid moths representing 131 species were collected, with many species displaying wide elevational distribution ranges. Moth α-diversity decreased monotonously, while the standardized effect size of mean pairwise phylogenetic distances (MPD) and phylogenetic diversity (PD) increased significantly with increasing elevation. At high elevations, the insect assemblages consisted largely of habitat generalists that were individually more phylogenetically distinct from co-occurring species than species in assemblages at lower altitudes. This could hint at higher speciation rates in more favourable low-elevation environments generating a species-rich geometrid assemblage, while exclusion of phylogenetically closely related species becomes increasingly important in shaping moth assemblages at higher elevations. Overall, it appears likely that high-elevation temperate moth assemblages are strongly resilient to environmental change, and that they contain a much larger proportion of the genetic diversity encountered at low-elevation assemblages in comparison to tropical geometrid communities.

Identifying biotic and abiotic determinants that shape the diversity patterns and composition of species assemblages represents one of the most challenging ecological issues of our time^1^,^2^. While the prevailing decrease in species richness with increasing latitude across most taxonomic groups is well-established^3^,^4^, the underlying causes for this and other diversity response patterns to environmental changes are poorly understood. Sanders and Rahbek^5^ suggest that detailed investigations of altitudinal species distribution patterns are the best approach to establish the ecological basis for species responses to environmental gradients. A monotonic decrease in biodiversity similar to that recorded for increasing latitudes has regularly been observed with increasing altitude. Nonetheless, other patterns such as hump-shaped responses and diversity plateaus have also been reported^6^,^7^,^8^,^9^.

While species surveys along altitudinal gradients provide us with information on current spatial species distribution patterns, a complementary phylogenetic analysis of species assemblages allows further key insights into spatio-temporal evolutionary pathways, as well as into phylogenetic and ecological traits^10^,^11^,^12^. Such an analysis enables us to gain important information about the factors underlying the current structure of species assemblages^13^,^14^,^15^. Evolving species and clades tend to retain some of their functional traits and niches, a phenomenon termed ‘niche conservatism’^1^. Such functional traits in turn form a strong base for understanding how assemblage structures are shaped by environmental factors^16^. Phylogenetic niche conservatism, when integrated into the general concept of niche conservatism, suggests that closely related species might share more similar ecological traits than species with more dissimilar phylogenies^1^.

Overall, studies into phylogenetic assemblage structures along altitudinal gradients, particularly at the level of individual families, are rare. Several studies have reported an increase in phylogenetic relatedness with increasing elevation. These include studies of trees^17^, butterflies^18^ and soil bacteria^19^. However, they focussed on high taxonomic levels, therefore including a wide variety of different families forming distinctive inherent phylogenetic clusters. As niche-differentiations and related ecological traits between different families are likely to be highly conservative over time^20^, a phylogenetic analysis combining numerous different families might underestimate the effects of environmental controls. An investigation of a species-rich family in this context may provide a different perspective in identifying the mechanisms shaping diversity patterns along environmental gradients.

In this study, we investigated the altitude-dependent species composition and phylogenetic structure in one of the most species-rich insect families, geometrid moths (Lepidoptera: Geometridae), as our target taxon. Geometrid moths are taxonomically well-known^21^, sensitive to environmental change^22^, and they can be surveyed readily on a range of different light sources^23^,^24^,^25^. Apart from a very wide range of plant species, the extensive range of food sources used by geometrid caterpillars even includes other insects^26^, and the wide ecological amplitude occupied by members of this family leads to their widespread distribution that regularly includes high latitude and altitude environments^27^,^28^,^29^,^30^,^31^.

The altitudinal diversity patterns of geometrid moths have been studied intensively in tropical forest ecosystems of South America^30^,^31^,^32^,^33^, Southeast Asia^34^,^35^,^36^ and Africa^27^,^37^. In contrast, altitudinal transect studies from temperate regions particularly in Asia are generally rare. Our study was carried out in the largest remaining mature temperate forest area of Northern China inside the Changbaishan Nature Reserve (CNR). Established in the 1960 s, this reserve contains one of the best-protected mature temperate forests in Asia^38^,^39^.

Similar to results from other taxa, previous studies of altitudinal α-diversity gradients in geometrid moths provide mixed outcomes, with a monotonous decrease^40^,^41^, hump-shaped distributions^42^ and other, more complicated distribution patterns^27^,^43^,^44^ being reported. In addition, Brehm, *et al*.^33^ recorded a clustering trend in phylogenetic relatedness with increasing elevation in geometrid assemblages from a South American tropical rainforest. This trend resulted in an overall decrease in phylogenetic diversity for a standardized species richness with increasing elevation, indicating that only a small group of strongly interrelated geometrid species occupied the high-elevation environments. Results from tropical regions might nonetheless not be easily transferable to temperate regions, not least since temperate species are commonly believed to have wider environmental tolerance ranges^45^,^46^.

Given the large prevailing gaps in knowledge and understanding of the diversity patterns in the highly species-rich insect taxa of temperate Asia, this study aimed to establish the spatial distribution of α-diversity for geometrid moths in the temperate forests of CNR along an altitudinal gradient, and to identify the drivers of the observed diversity changes. In response to environmental conditions becoming increasingly less favourable, we hypothesized that α-diversity in geometrid moths would decrease monotonously with increasing elevation. Our second aim was to examine patterns of phylogenetic relatedness in geometrid moth assemblages along the altitudinal gradient to obtain insights into underlying evolutionary and ecological processes. In this context, we hypothesized that geometrid species at high elevations would show strong phylogenetic relatedness and traits allowing them to adapt to the less favourable, more extreme environmental conditions in comparison to conditions experienced by species at lower elevations. We therefore hypothesised that the phylogenetic diversity for a standardized species richness would decrease with increasing elevation.

## Results

In total, we caught 9285 geometrid moths. Of these, 1072 specimens (11.5%) were so badly damaged due chiefly to rainfall events in sampling nights that identification was deemed impossible. The remaining 8213 individuals were initially divided into 156 morpho-species. Apart from 1 rare species (2 individuals), all remaining 155 morphospecies formed the basis for DNA sequencing, and the pre-estimated species number was subsequently reduced to 130 molecular operational taxonomic units (MOTUs). Overall, the 131 putative species present in our samples represented five subfamilies; Ennominae, Geometrinae, Larentiinae, Orthostixinae and Sterrhinae (note: the validity of Orthostixinae at subfamily level is controversial, it was supported in the molecular phylogenetic analyses of Yamamoto and Sota^47^ and Sihvonen, *et al*.^48^, but questioned by Sihvonen, *et al*.^49^).

**Figure 1:**
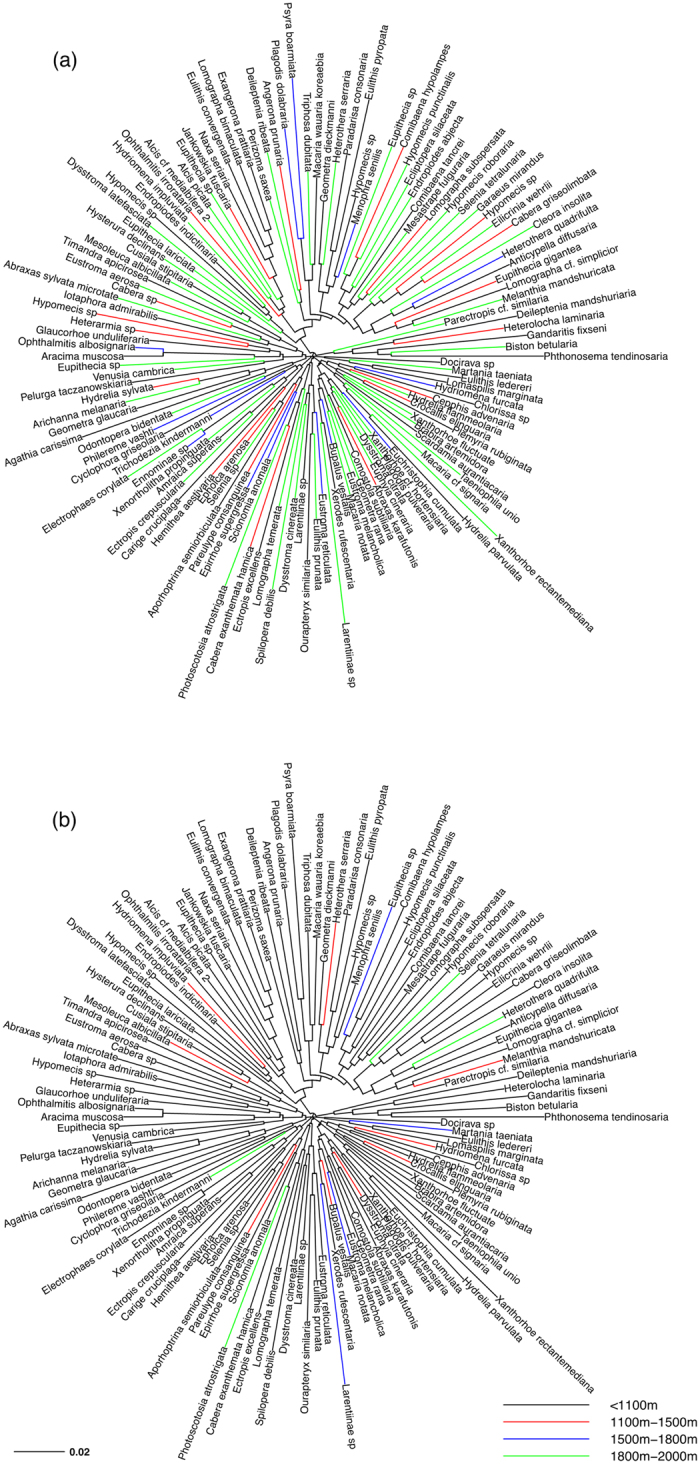
Neighbour-joining trees (COI 5’ data, based on K2P distance) of geometrids on Changbai Mountain. Different colours of branch tips illustrate where species reach their maximum (**a**) and minimum (**b**) altitudinal limit.

The phylogenetic trees (Fig. 1) show that a large number of species occurred across a wide altitudinal range, with 44 species recorded in the entire forest area covered by the altitudinal transect. Furthermore, species with more limited distribution ranges were distributed across the entire taxonomic tree.

**Figure 2:**
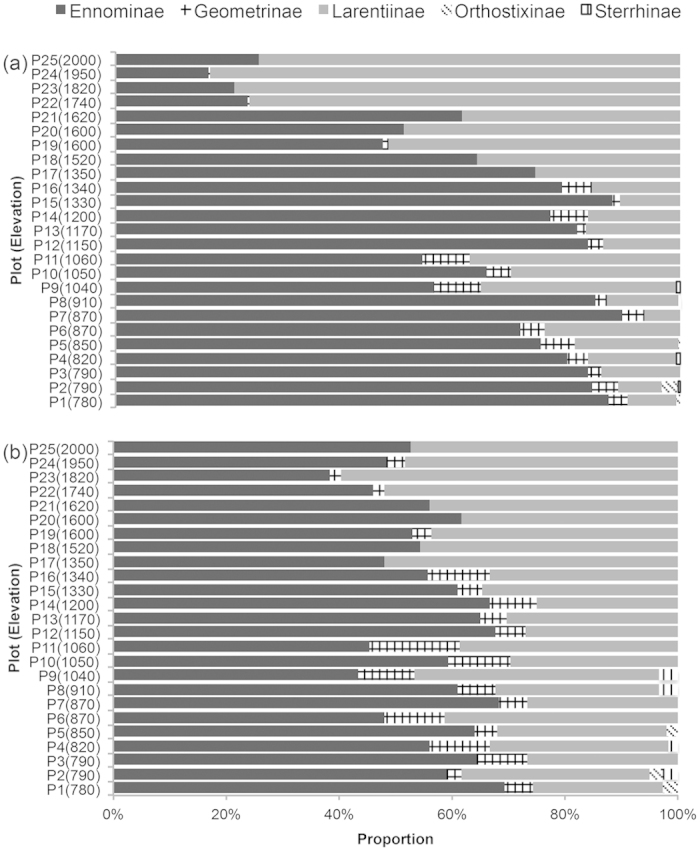
Proportion of subfamilies abundance (**a**) and species richness (**b**) at each sampling plot, ordered by elevation.

In relation to diversity patterns of individual subfamilies, Ennominae dominated at low elevations, while Larentiinae became increasingly dominant above 1600 m. Orthostixinae and Sterrhinae only occurred at elevations below 1100 m (Fig. 2). Along with the increase in elevation, the proportion of both Ennominae and Geometridae decreased in relation to both, overall abundance (Pearson correlation: r = −0.82 and r = −0.66, *P *< 0.001 for both case) and species richness (r = −0.48, *P* = 0.013, and r = −0.57, *P* = 0.003, respectively), while Larentiinae showed a significant increase in their proportional representation across the samples (r = 0.86 for abundance and r = 0.75 for richness, both at *P *< 0.001).

**Figure 3:**
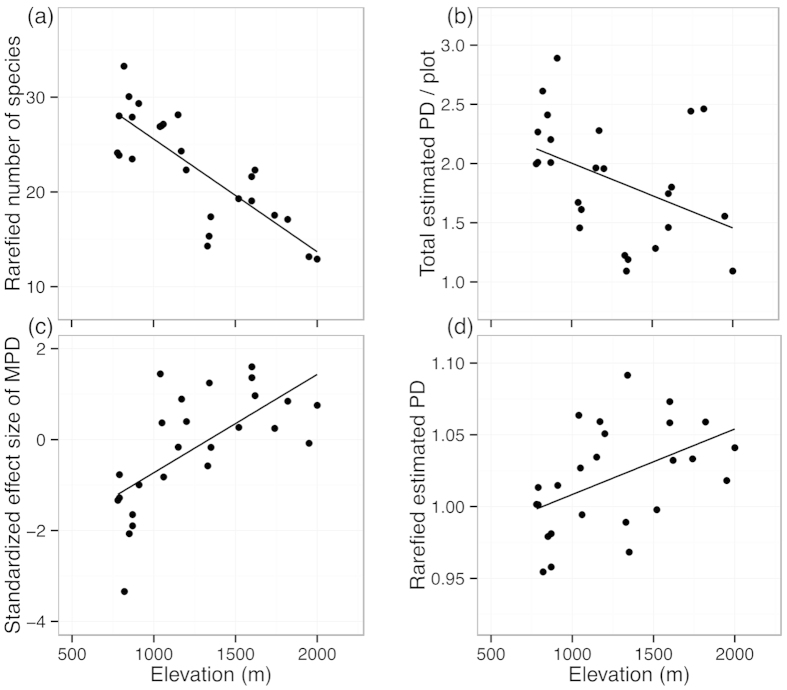
Rarefied number of species (**a**), total estimated phylogenetic diversity (**b**), standardized effect size of mean pairwise phylogenetic distance (MPD) (**c**) and rarefied estimated phylogenetic diversity (n = 18) (**d**) for geometrid moths with changing elevation.

The overall rarefied species number of geometrid moths decreased significantly with increasing altitude (Pearson correlation, r = −0.81, *P *< 0.001, Fig. 3a). The overall estimated PD of each plot was linearly correlated with the total number of recorded species (Pearson correlation, r = 0.99, *P *< 0.001), hence also showing a significant decrease with increasing elevation (r = −0.42, *P* = 0.034, Fig. 3b). In contrast, the standardized effect size of MPD increased significantly with increasing elevation over the transect (r = 0.66, *P *< 0.001, Fig. 3c). Species showed a trend to form phylogenetic clusters (mean value = −1.74) at elevations below 1000 m asl., a mixed pattern between 1000 m and 1500 m (mean value = 0.28), and a trend to over-dispersion above 1500 m (mean value = 0.70). When standardizing the PD values for the minimum number of recorded species across the plots (n = 18), a significant positive correlation with elevation was established (r = 0.47, *P* = 0.016, Fig. 3d), again indicating an overall positive correlation between the degree of over-dispersion in moth assemblages and altitude.

## Discussion

In accordance with our first hypothesis, the α-diversity of geometrid moths decreased significantly and monotonously with increasing elevation across the entire altitudinal gradient. This result is in line with previous observations in the CNR area of geometrid moths by Chen *et al*.^41^, and of other insect taxa such as noctuid moths^40^ and ground beetles^50^, as well as for plant species^17^. The results suggest that only relatively few geometrid species can live in the cold conditions occurring at high altitude forests on Changbai Mountain. The decreasing geometrid species richness can also be related to the increased limitation of available resources linked to the decrease both in the overall plant diversity and in the area of altitudinal bands from the base to the top of the mountain^51^,^52^.

The observed proportional decrease in Ennominae and increase in Larentiinae along the altitudinal gradient are consistent with previous studies from South America^33^, Southeast Asia^42^ and East Africa^27^. The increased proportion of Larentiinae with increased elevation reflects particularly strong adaptations to cooler and wetter environmental conditions in comparison to other geometrid subfamilies^53^. Members of the Sterrhinae in this study showed a clear elevational boundary well below the forest line, with members of this family not encountered above 1100 m. This again mirrors findings by Axmacher, *et al*.^27^, who reported that no Sterrhinae were present above 2600 m in the afro-tropical forests of Mt Kilimanjaro, indicating that Sterrhinae are particularly sensitive to the severe climatic conditions encountered at high-mountain environments.

In direct contradiction to our second hypothesis, a surprising key finding of this study is that both the average phylogenetic diversity and the phylogenetic diversity for a standardized species number increase significantly with increasing elevation. The species in the smaller species pool encountered at high elevations are therefore genetically significantly more distinct than species in assemblages at lower elevations, indicating an aggregating of phylogenetic lineages in low and an over-dispersion in high-elevation forest moth assemblages. This result strongly contrasts observations of tropical geometrid assemblages by Brehm, *et al*.^33^, who reported a trend for over-dispersion at low elevations and a trend for phylogenetic clustering at high elevations. Given the much higher overall species richness in tropical Andean forests and the more complex α-diversity patterns with changing elevation^43^, the contrast might be partly explained by the general differences between tropical and temperate geometrid assemblages and species pools. Due to the much greater uniformity of environmental conditions in tropical forests, species in these regions can have much narrower distribution ranges associated with narrower niche tolerances in comparison to species in temperate regions^46^. Our results indeed support this assumption, given the large number of species with a wide altitudinal distribution range recorded from our study area. Most of these species must be assumed to be habitat and host-plant generalists, given their wide distribution across the different forest zones with their distinct differences not only in the tree cover, but also in the undergrowth vegetation^54^. As our study area is covered in continuous forest vegetation and lacks strong physical barrier, these forest habitat and host-plant generalist moth species can easily disperse across the entire mountain range. Furthermore, our results indicate that cold tolerance in geometrid moths on Changbai mountain did not originate from a small number of basal lineages as observed in the tropics. In contrast, it appears that widely unrelated sets of habitat generalists have obtained this ability independently throughout their evolutionary past.

Our finding also stand in contrast to the increased phylogenetic relatedness with increasing elevation observed in angiosperms from our study region^17^. This might at first seem surprising, given that moths are closely linked to plants as herbivores and pollinators. Nonetheless, since Qian *et al*.^17^ focussed on a higher taxonomic level where phylogenetic patterns and associated traits are expected to be differentiated much more strongly than within species of a single family, results are not directly comparable. Shorter life cycles, higher fecundity and mobility in insects may also result in general differences between the phylogenetic patterns of insects and plants along spatial and environmental gradients. While niche theory suggests strong positive links between the α-diversity of herbivorous geometrid moths and plants, the existence of such positive links has furthermore rarely been recorded, with weak, non-significant or even significantly negative correlations reported from a number of field studies^27^,^44^. The underlying lack of similarities in spatial α-diversity patterns of moths and plants was also observed from our study area (Zou, unpublished data). Overall, our results indicate that host-pant generalists dominate our temperate geometrid moth assemblages.

Local species pools are generally directly determined by the processes of speciation, extinction and dispersal^55^,^56^,^57^. These determinants are strongly related to inter- and intra-specific interactions as well as to environmental conditions. Under natural conditions, the actual composition of species assemblages is widely determined by both environmental filtering and interspecific repulsion^19^,^58^,^59^. In cases where local species pools are dominated by clusters of phylogenetically closely related species, their sharing of similar environmental niches suggests that repulsion in these clusters is only a minor determinant for the assemblage composition. In these cases, evolution will have favoured the specific traits present in these clusters that render them well-adapted to the local environmental settings. At low elevations, warm temperatures and a large amount and diversity of available resources are associated with an increased availability of niche space. Our results indicate that in combination with an intense intra-specific competition, these environmental conditions have resulted in higher speciation rates that have led to the existence of a greater diversity in phylogenetically closely related geometrid species in comparison to assemblages at higher elevations^60^,^61^. Overall, it must be remembered that a large proportion of the regional geometrid species pool consists of environmental generalists that are able to survive across the environmental gradient, but with distinctly more low-elevation than high-elevation specialists being present in the samples. With increasing elevation, exclusive competition appears to become increasingly important for closely related sets of geometrid moth species. This pattern can be linked to the combination of increasingly hash environmental conditions, a reduction in available resources and the decrease in overall available area, and it explains the observed formation of high-elevation assemblages consisting of fewer, phylogenetically more distinct species.

Despite the decrease in species richness and the associated overall phylogenetic information contained in the moth assemblages with increasing elevation in this temperate forest ecosystem, their increased phylogenetic distinctiveness actually indicates a high diversity of the assemblages from a genetic perspective. This is likely linked to a strong resilience of the overall high-elevation assemblage towards environmental changes, an assumption further supported by the large proportion (69%) of environmental generalist species in this assemblage that were recorded over the entire environmental gradient. Of the 54 geometrid species encountered between 1800 and 2000 m, only four were restricted to this elevational band. Our results also provide support to the assumption that species in temperate geometrid moth assemblages are adapted to much wider ranges of environmental conditions throughout their phylogenetic lineages in comparison to tropical assemblages where the ability to survive in high-altitude environments appears much more restricted to a limited number of phylogenetic lineages.

## Methods

### Study area and sampling plots

The study was conducted on the northern slopes of Changbai Mountain (E127°43′–128°16′; N41°41′–42°51′), Jilin Province, Northeast China. The natural vegetation in this area can be classified as cool-temperate moist forest based on Holdridge’s^62^ life zone system^63^. A linear decrease in temperature with increasing elevation is accompanied by a general increase in precipitation along the entire mountain range^17^. Along the altitudinal gradient, distinct forest zones can be distinguished: mixed coniferous and broad-leaved forests at the mountain base (700 m–1100 m), followed with increasing elevation by mixed coniferous forests (1100 m–1500 m) and sub-alpine mixed coniferous forests (1500 m–1800 m), with birch forests (1800 m–2100 m) forming the upper forest boundary^54^,^64^,^65^.

### Sampling of geometrid moths

We selected 25 sampling plots located in the aforementioned forest types along an altitudinal gradient ranging from 700 m to 2000 m. Geometrid moths were sampled in automatic light traps. Sampling was conducted between 19:30 and 22:30, when geometrid moths are most active, with each plot sampled once each month in July and August in 2011 and in June 2012.

All specimens were first sorted to morpho-species. Subsequently, samples of the majority of morpho-species were sent to the Canadian Centre for DNA Barcoding to analyse the interspecific genetic dissimilarity as a proxy for phylogenetic distances.

### Data analysis

As the number of observed species usually provides only a poor representation of α-diversity in mobile insects^66^, we chose Hurlbert rarefaction^67^ for the comparison of the α-diversity in geometrid moth assemblages between sampling plots. These rarefaction techniques have proven robust in comparing diversity values in a wide range of ecological studies^68^,^69^,^70^.

For species identification, the first step was based on distinguishing morpho-species based on their wing patterns. In a second step, 152 selected specimens representing these morpho-species were submitted to DNA barcoding, which allowed discrimination to molecular operational taxonomic units (‘MOTUs’) based on a sequence divergence threshold of 2%^71^,^72^ using the Kimura 2-parameter (K2P) distance^73^. This also roughly corresponds to the BIN system implemented on the Barcode of Life Data System (BOLD) database^74^. If individuals of a species varied below the 2% threshold, the sequence with the shortest distance to the other respective species was included in the subsequent analysis^33^. DNA barcoding allowed species identification and attribution to a Linnean binomen in 127 of the 152 MOTUs. These were supplemented by 3 MOTUs of species that we identified, but where we then used data already stored on BOLD for our subsequent analysis rather than sending specimens for barcoding ourselves. Although we acknowledge that some distinct species may share very similar COI sequences below the 2% threshold, we believe that this effect will be negligible for our limited samples from one small study region. Species were then further identified based on the comparison of MOTU information from BOLD database, where all our sequence data were also deposited.

Based on the K2P distance, the expected phylogenetic diversity (PD, sum of total phylogenetic branch length) for all species recorded at individual plots was then estimated using Faith’s index^75^. Additionally, the standardized effect size of the mean pairwise phylogenetic distance (MPD) was calculated to evaluate whether the respective species assemblage is clustered or over-dispersed across the phylogenetic tree. In this approach, a low and negative value indicates clustering on the phylogenetic tree, whereas high and positive values indicate an evenly or over-dispersed phylogenetic pattern^76^. The value of this index represents the reverese of the relatedness index^77^,^78^,^79^. It was calculated based on 1000 random iterations drawn from the recorded geometrid species pool.

All calculations and statistical analysis were conducted in R^80^. We used the ‘vegan’ package^81^ to calculate rarefactions, ‘Picante’^77^ for the calculation of Faith’s PD and MPD and ‘ape’^82^ for calculations of phylogenetic distances.

## Additional Information

**How to cite this article**: Zou, Y. *et al*. High phylogenetic diversity is preserved in species-poor high-elevation temperate moth assemblages. *Sci. Rep*. **6**, 23045; doi: 10.1038/srep23045 (2016).
